# Heat Shock Protein 70 and The Risk of Multiple Sclerosis in The Iranian Population

**DOI:** 10.22074/cellj.2019.5620

**Published:** 2018-08-07

**Authors:** Seyedeh Parisa Chavoshi Tarzjani, Seyed Abolhassan Shahzadeh Fazeli, Mohammad Hossein Sanati, Seyed Massood Nabavi

**Affiliations:** 1 Department of Biological Sciences, Tehran North Branch, Islamic Azad University, Tehran, Iran; 2 Department of Genetics, Reproductive Biomedicine Research Center, Royan Institute for Reproductive Biomedicine, ACECR, Tehran, Iran; 3 Iranian Biological Resource Center (IBRC), ACECR, Tehran, Iran; 4 National Institute for Genetic Engineering and Biotechnology, Tehran, Iran; 5 Department of Neurology, School of Medicine, Mostafa Khomeini Hospital, Shahed University, Tehran, Iran

**Keywords:** *HSP70*, Iranian, Multiple Sclerosis, Polymorphism

## Abstract

Multiple sclerosis (MS) is a chronic disease of the central nervous system and one of the most common causes of neurological disability among those aged 20-40 years, particularly in women. Major histocompatibility complex (MHC) Class II genes are known to be involved in the development of MS. One of the important groups of this complex is the HSP gene family, especially *HSP70*, which is induced under stress conditions. The aim of the present case-control study was to determine the association between the heat shock protein 70 (*HSP70*) and risk of MS in Iranian patients by genotyping the rs1061581 gene polymorphism. A total of 50 relapsing-remitting MS (RRMS) patients and 50 healthy control subjects were considered for this study. Genotyping was performed by the polymerase chain reaction-restriction fragment length polymorphism (PCR- RFLP) method. PCR-RFLP results of twenty-five randomly selected samples were confirmed by DNA sequencing. Genotypic and allelic distributions were compared between the case and control groups. We observed no significant difference in the distribution of rs1061581 genotype and allele frequencies between RRMS patients and controls. In addition, there was no association between the *HSP70* gene polymorphism and the clinical variables in the case group. Our data indicate that *HSP70*, in particular rs1061581, is unlikely to be involved in the susceptibility to or the severity of RRMS in Iranian patients. Further large prospective studies are required to confirm these findings.

 Multiple Sclerosis (MS) is an inflammatory, neurodegenerative, chronic disease of the central nervous system (CNS) (1, 2). MS is one of the most common causes of neurological disability in young adults aged between 20 and 40 years, which is more frequent in women. MS leads to symptoms such as blurred vision, muscle weakness and spasm (3). Relapsing-Remitting MS (RRMS), the most frequent clinical form of MS, accounts for approximately 80 to 85% of MS patients (4). MS is influenced by environmental risk factors including smoking, Epstein-Barr virus (EBV) infection and vitamin D/ultraviolet (UV) deficiency, however, genetic factors also play an important role in this disease (1, 5). The most important gene conferring susceptibility to MS (although with a weak effect) is the MHC class II (HLADRB1*1501 allele) locus (6). Human leukocyte antigen (HLA) locus is located on the short arm of chromosome 6 with one of its gene complexes being the heat shock protein (HSP) gene family (7).

HSPs are a group of phylogenetically conserved proteins found in all prokaryotic and eukaryotic cells (8). Their expression dramatically increases under conditions of stress including free radicals, toxic metal ion exposure, heat and hypoxia (9). These proteins are named according to their molecular weight, which ranges from 17 kDa to more than 100 kDa, are classified into six families, namely the *HSP100, HSP90, *HSP70*, HSP60, HSP40* and the small HSP families (10). Recently, there have been reports regarding the association between *HSP70* gene polymorphisms and different human autoimmune diseases. In insulin-dependent diabetes mellitus (11), celiac disease (CD) (12), long QT syndrome (LQTS) (13) and sarcoidosis (14), significant differences have been observed in the distribution of *HSP70* genotype or allele frequencies between patients and controls. Moreover, its beneficial effect in Alzheimer’s and Parkinson’s diseases has been suggested (15, 16). 

*HSP70* have two physiological neuroprotective roles. In specific, they act as molecular chaperones that assist the proper folding of newly synthesized proteins, preventing protein aggregation, and degrading unstable and misfolded proteins (17). *HSP70* may also act as a cytokine by stimulating a pro-inflammatory signal transduction cascade in monocytes (18). It has been proposed that in MS patients, overexpression of *HSP70* proteins can protect the CNS from inflammation so that the CNS can help towards myelin repair (19). Three genes encoding *HSP70* (*HSPA1A, HSPA1B,* and *HSP-HOM*) are located within the HLA class III subregion (chromosome 6p21.3) with HSPA1A and HSPA1B being 99% identical (20).

The association of *HSP70* gene polymorphisms and MS has been investigated based on the 1267 A/G polymorphism in the *HSP70*-2 coding region and the 2437 T/C polymorphism in the *HSP70*-hom coding region in Canadian MS patients (21) while the promoter region polymorphism of *HSP70*1has been analysed in Italian MS patients (22). Previous studies have shown that *HSP70*-2 gene polymorphisms and *HSP70*-2 protein level expression are significantly associated with the presence of MS in Italian patients (23). On the other hand, no association between *HSP70* gene polymorphisms and susceptibility to or the severity of MS was observed in Japanese patients (24). In addition, an association has been reported between a *HSP70* gene polymorphism (rs1061581) and noise-induced hearing loss (25), a risk association of these polymorphisms with coronary artery disease (26). 

Hence, the present case-control study was undertaken to determine the association of this *HSP70* gene polymorphism and susceptibility to MS in the Iranian population. For the present case-control study, a total of 50 RRMS patients between 20-40 years of age were selected for this study. A total of 50 healthy individuals matched for age and sex formed the control group. At the time of blood sample collection, all the controls had been assessed to be free from any kind of disorders, whether physical or mental. The subjects were included under the study with their written informed consent. All of the patients and controls were of Iranian origin.

Whole blood was collected by venipuncture in tubes containing EDTA. Human genomic DNA was obtained from 200 µl of whole blood using the Gene All DNA Blood Mini Kit (Exgene Clinic SV, Korea) according to the manufacturer’s instructions. The concentration and purity of DNA samples were determined by spectrophotometric analysis. The *HSP70* gene polymorphism was genotyped using polymerase chain reaction-restricted fragment length polymorphism (PCR-RFLP). The primer pair for this single nucleotide polymorphism (SNP) was designed using the Perlprimer software (Table 1). 

**Table 1:** Primers and restriction enzymes used for genotyping the HSP70 (1053G>A) gene polymorphism


Polymorphism	Primer (5ˊ-3ˊ)	Size	Enzyme

1053 G>A	F: CATCGACTTCTACACGTCCA	1117 bp	PstI
	R: ATACTAGGAAATGCAAAGTCT		


The PCR cycling conditions were an initial melting step of 3 minutes at 95°C, followed by 35 cycles of 30 seconds of denaturation at 95°C, 30 seconds of annealing at 58.1°C and 1 minute extension at 72°C, and a final elongation step of 5 minutes at 72°C. The genotyping of this SNP (1053 G>A in the *HSP70* coding region) was undertaken by digesting the PCR products with the PstI restriction enzyme (two-hour incubation at 37°C). Both after amplification and digestion, the presence of products was confirmed by agarose gel electrophoresis (Figes.1, 2).

The DNA bands of 181 bp and 936 bp were observed after digestion. Asingle band of 1117 bp represented the AA(variant) genotype. Two bands of 181 bp and 936 bp represented the GG (ancestral) genotype and all three bands represented the AG (heterozygous) genotype. To confirm the results of the PCR-RFLP method, twenty-five randomly selected PCR products were sequenced. The resulting sequences were then analyzed for genotypes using the FinchTV software. To assess the association between the examined polymorphism and RRMS we performed logistic regression analysis and adjusted for sex and age. Adjusted odds ratios (OR) with 95% confidence intervals (95% CI) were derived and used as the measure of effect.

The allele and genotype frequencies amongst cases and controls were compared by the Chi-square test. All statistical analyses were undertaken in SPSS (SPSS Inc., Chicago, IL, USA). The mean age at the time of collection of RRMS patients and controls were 33 ± 1 and 34 ± 1 years. The RRMS patient group consisted of 13(26.0%) males and 37(74.0%) females while the control group consisted of 14 (28.0%) males and 36 (72.0%) females. The amplified PCR products of the *HSP70* gene observed on a 1% agarose gel are shown in Figure 1 and digested products are shown in Figure 2. The distribution of allele and genotype frequencies of the *HSP70* (1053 G>A) polymorphism is shown in Table 2. Analysis of sequencing confirmed the results of PCR-RFLP. 

**Fig.1:**
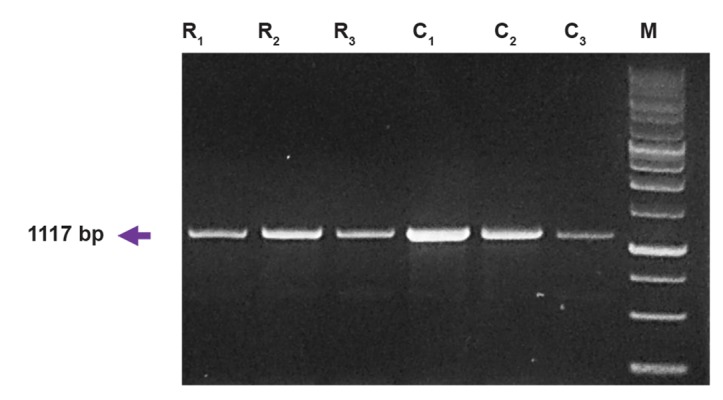
Polymerase chain reaction amplification of the *HSP70* gene. Lane M represents DNA ladder (1 Kb); lane R_1_, R_2_ and R_3_ represents relapsing- remitting multiple sclerosis (RRMS) patients; lane C_1_, C_2_ and C_3_ represents healthy control individuals.

**Fig.2:**
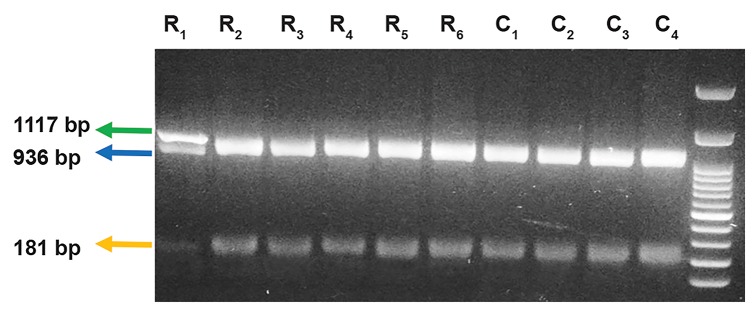
Agarose gel electrophoresis of restriction fragment length polymorphism (RFLP) products of *HSP70* fragments containing the 1053 G>A gene polymorphism. Loading sequence: 100 bp ladder. Lane R_1_- R_6_ [relapsing-remitting multiple sclerosis (RRMS) patients] and lane C_1_- C_4_ (healthy control): GG genotype (wild type). Lane 1(R1): AG genotype (heterozygous).

Genotypic frequencies of *HSP70* gene pointed to a non-significant association between polymorphism (AA/AG/GG) were observed at 0, genotype and presence of RRMS (sex and age6.0, 94.0% in RRMS patients and at 0, 2.0, 98.0% adjusted OR of 3.12 (0.31-31.53), P=0.33, X^2^=0.182). in healthy controls respectively. The demographic Logistic regression analysis adjusted by sex and and clinical characteristics of RRMS patients age indicatedno significant association between the and controls are presented in Table 3. The logistic examined polymorphism and RRMS (P value_sex_=0.88, regression allelic additive model (crude and adjusted) P value_age_ =0.58, Table 4).

**Table 2:** Genotype and allelic frequencies of *HSP70* (1053 G>A) gene polymorphism in MS patients and controls


HSP70 (1053 G>A)		Cases	Controls	Adjusted OR (95% CI)	P value
		n (%)	n (%)		

**Genotype**					
	GG	49 (98.0)	47 (94.0)	3.12 (0.31-31.53)	0.33
	AG	1 (2.0)	3 (6.0)		
	AA	0 (0)	0 (0)		
**Allele**					
	G	99	97	0.32 (0.32-3.18)	0.617
	A	1	3		


MS; Multiple sclerosis, OR; Odds ratios, and CI; Confidence intervals.

**Table 3:** Demographic and clinical characteristics of RRMS patients and controls


Characteristic		Patient	Control
	(Total)	n=50	n=50
		n (%)	n (%)

Gender			
	Male	13 (48.1)	14 (51.9)
	Female	37 (50.7)	36 (49.3)
Age (Y)			
	<30	24 (48.0)	23 (46.0)
	>30	26 (52.0)	27 (54.0)
Smoking			
	Smokers	10 (20)	7 (14)
	Non-smokers	40 (80)	43 (86)
Daily intake of vitamin D		45 (90)	_
Change in EDSS		0-2	_


EDSS; Expanded disability status scale.

**Table 4:** Conditional logistic regression analysis


Characteristic		MS		SE	P value (Crude)	OR (95% CI)(Crude)	P value (adjusted)	OR (95% CI)(adjusted)
		No	Yes					
		n (%)	n (%)					

Gender								
	Male	14 (51.9)	13 (48.1)	0.46	0.82	0.37 (0.37-2.19)	0.88	1.07 (0.44 -2.6)
	Female	36 (49.3)	37 (50.7)					
Age								
	<30	23 (46.0)	24 (48.0)	0.13	0.02	0.84 (0.71-0.99)	0.58	1.02 (0.95-1.09)
	≤30	27 (54.0)	26 (52.0)					


MS; Multiple sclerosis, OR; Odds ratio, and CI; Confidence interval.

We found no significant difference between RRMS patients and controls in the Iranian population based on the *HSP70* variant (P>0.05). The overexpression of *HSP70* in MS lesions might protect CNS cells against the inflammatory environment that is typical of the stress conditions (9). As a result, therapeutic strategies focusing on HSP up-regulation have been proposed for different neuropathologies that generally are characterized by misfolded protein aggregation (27). Although the release of *HSP70* in Alzheimer’s and Parkinson’s diseases leads to a reduction in misfolded proteins, it exacerbates the immune response in MS by acting as an adjuvant for myelin peptides and as a pro-inflammatory cytokine. In addition, *HSP70* can contribute to autoimmunity (9, 27). High levels of autoantibodies against *HSP70* have been found in MS patients (28). Moreover, *HSP70*-MBP in the brain tissue of MS patients has been proposed as possible target autoantigens in MS (29). Recently, a strong association between *HSP70*-hom gene polymorphism and protein expression has been reported in MS (30).

We therefore focused on the study of *HSP70*, examining the role of the 1053 G>A (rs1061581) polymorphism in RRMS patients. SNPs located in the coding region of the *HSP70* gene causes a synonymous mutation (Q351), which does not change amino acid sequence. The lack of association observed in this study is in accordance with the findings from Japan (24), however, it was different from that in Italy (23). These differences may be due to the ethnic variations of *HSP70* gene polymorphisms.

The previous study in Japan indicated that *HSP70* gene polymorphisms were not associated with susceptibility to MS in the Japanese MS population (24). In addition, Ramachandran and Bell reported that there were no significant differences in genotype frequencies between MS patients and controls in either *HSP70*-2 or *HSP70*hom, and the gene polymorphisms of *HSP70*-2 and *HSP70*- hom did not increase susceptibility to MS (21). Cascino et al. (22) reported no significant difference between the MS patient group and the control group in the promoter region polymorphism of *HSP70*-1. In contrast, a study has shown that a *HSP70*-2 gene polymorphism and *HSP70*-2 protein level expression are significantly associated in Italian MS patients (23). The lack of replication in the Iranian population may be due to the low frequency of the variant compared with the other studies mentioned.

The *HSP70* gene family consists of multiple highly homologous genes and the effect of one SNP in one *HSP70* gene may thus be limited. Hence, haplotype analysis of multiple SNPs in *HSP70* genes would be needed in future studies. There is also the possibility that *HSP70* gene polymorphisms are a susceptibility factor to MS in an ethnic-specific manner.

## Conclusion

We conclude that rs1061581 polymorphism in *HSP70* is unlikely to be associated with the development of RRMS in the Iranian population. However, the small sample size of the groups studied here warrant further analysis.
